# Supplementary material for: History of Polish Canidae (Carnivora, Mammalia) and Their Biochronological Implications on the Eurasian Background
Source: Genes (Basel). 2023 Feb 21;14(3):539. doi: 10.3390/genes14030539 (PMC10048199; doi:10.3390/genes14030539)
Supplement: Supplementary file 1 [file genes-14-00539-s001.zip › genes-2087133-supplementary.pdf]

List of Polish sites with canid remains in chronological order

1. Site: Podlesice (4.9-4.2 myr).
Canid species: Canidae indet. (jackal size).
Sources: this paper.

2. Site: Mała Cave, layers 4-5 (4.9-4.2 myr).
Canid species: Canidae indet. (jackal size).
Sources: this paper.

3. Site: Węże 1 (3.8-3.4 myr).
Canid species: *Eucyon odessanus*, *Nyctereutes donnezani*.
Sources: [1–8]

4. Site: Ewy Cave (3.8-3.4 myr).
Canid species: Canidae indet (fox size).
Sources: this paper.

5. Site: Raciszyn 1 (3.8-3.4 myr).
Canid species: Canidae indet (fox size).
Sources: this paper.

6. Site: Mokra 1 (3.8-3.4 myr).
Canid species: Canidae indet (fox size).
Sources: this paper.

7. Site: Zalesiaki 1B (3.8-3.2 myr).
Canid species: Canidae indet (fox size).
Sources: this paper.

8. Site: Węże 2 (2.9-2.6 myr).
Canid species: *Canis* cf. *etruscus*, *Nyctereutes* sp.
Sources: this paper.

9. Site: Rębielice Królewskie 1A (2.4-2.2 myr).
Canid species: *Lycaon falconeri*, *Canis etruscus*.
Sources: this paper.

10. Site: Mokra 2 (2.6-2.2 myr).
Canid species: *Vulpes* sp.
Sources: this paper.

11. Site: Zamkowa Dolna Cave, fauna A (2.4-2.2 myr).
Canid species: *Lycaon falconeri*, *Canis etruscus*, *Vulpes* cf. *alopecoides*.
Sources: this paper.

12. Site: Kielniki 3B (2.2-1.8 myr).
Canid species: *Canis etruscus*, *Vulpes* cf. *alopecoides*.
Sources: this paper.

13. Site: Kadzielnia (2.2-1.8 myr).
Canid species: *Canis etruscus*, *Vulpes praeglacialis*.
Sources: this paper.

14. Site: Kamyk (1.9-1.8 myr).
Canid species: *Canis etruscus*, *Vulpes praeglacialis*.
Sources: [9–11].

15. Site: Żabia Cave (1.7-1.5 myr).
Canid species: *Lycaon lycaonoides*, *Canis mosbachensis*, *Vulpes praeglacialis*.
Sources: [12–13].

12. Site: Kielniki 3A (1.5-1.3 myr).
Canid species: *Canis mosbachensis*, *Vulpes praeglacialis*.
Sources: this paper.

16. Site: Zalesiaki 1A (1.1-0.9 myr).
Canid species: *Canis mosbachensis*, *Vulpes praeglacialis*.
Sources: [9–11].

12. Site: Kielniki 1 (1.1-0.8 myr).
Canid species: *Lycaon lycaonoides*, *Canis mosbachensis*, *Vulpes praeglacialis*.
Sources: [9-11].

11. Site: Zamkowa Dolna Cave, fauna C (1.0-0.8 myr).
Canid species: *Canis mosbachensis*, *Vulpes praeglacialis*.
Sources: this paper.

17. Site: Przymiłowice (MIS 22-17, 0.9-0.7 myr).
Canid species: *Canis mosbachensis*, *Vulpes praeglacialis*.
Sources: this paper.

18. Site: Kozi Grzbiet (MIS 19-17).
Canid species: *Lycaon lycaonoides*, *Canis mosbachensis*, *Vulpes praeglacialis*.
Sources: [9–11,14].

19. Site: Sitkówka (MIS 19-17).
Canid species: *Canis mosbachensis*.
Sources: this paper.

20. Site: Południowa Cave (MIS 17-15).
Canid species: *Lycaon lycaonoides*, *Canis mosbachensis*, *Vulpes vulpes angustidens*, *Vulpes praeglacialis*.
Sources: [15–21].

9. Site: Rębielice Królewskie 2 (MIS 15-13).
Canid species: *Canis mosbachensis*, *Vulpes vulpes angustidens*, *Vulpes praeglacialis*.
Sources: this paper.

21. Site: Tunel Wielki Cave (MIS 14-12).
Canid species: *Lycaon lycaonoides*, *Canis mosbachensis*, *Cuon alpinus priscus*, *Vulpes vulpes* ssp.
Sources: [22].

22. Site: Draby 3 (MIS 11).
Canid species: *Lycaon lycaonoides*, *Canis mosbachensis*, *Vulpes vulpes*.
Sources: [23].

23. Site: Biśnik Cave, layers 19ad-19 (MIS 9).
Canid species: *Canis lupus lunellensis*, *Cuon alpinus priscus*, *Vulpes vulpes*, *Vulpes lagopus*.
Sources: [23–24].

24. Site: Deszczowa Cave, fauna 1 (MIS 9-8?).
Canid species: *Canis lupus lunellensis*, *Cuon alpinus fossilis*, *Vulpes vulpes*, *Vulpes* cf. *praeglacialis*.
Sources: [23,25–27]

25. Site: Wschodnia Cave (MIS 8-7).
Canid species: *Canis lupus* ssp., *Cuon alpinus fossilis*, *Vulpes vulpes*, *Vulpes lagopus*.
Sources: [23].

26. Site: Naciekowa Cave (MIS 8).
Canid species: *Canis lupus* ssp., *Vulpes vulpes*.
Sources: [28].

23. Site: Biśnik Cave, layers 18-14 (MIS 7-6).
Canid species: *Canis lupus* ssp., *Cuon alpinus fossilis*, *Vulpes vulpes*, *Vulpes lagopus*.
Sources: [23,28].

27. Site: Wierzchowska Górna Cave (MIS 7-1).
Canid species: *Canis lupus* ssp., *Vulpes vulpes*, *Vulpes lagopus*.
Sources: [2,29–34].

28. Site: Nietoperzowa Cave (MIS 6-1).
Canid species: *Canis lupus spelaeus*, *Canis lupus lupus*, *Vulpes vulpes*, *Vulpes lagopus*.
Sources: [9,11,26,34–37].

29. Site: Komarowa Cave (MIS 6-1).
Canid species: *Canis lupus spelaeus*, *Canis lupus lupus*, *Vulpes vulpes*, *Vulpes lagopus*.
Sources: [26–27].

30. Site: Dziadowa Skała Cave (MIS 5e-1).
Canid species: *Canis lupus lupus*, *Vulpes vulpes*, *Vulpes lagopus*.
Sources: [46].

31. Site: Niedźwiedzia Cave (MIS 5-1).
Canid species: *Canis lupus spelaeus*, *Canis lupus lupus*, *Vulpes vulpes*, *Vulpes lagopus*.
Sources: [38–45].

32. Site: Mamutowa Cave (MIS 5-2).
Canid species: *Canis lupus spelaeus*, *Canis lupus lupus*, *Vulpes vulpes*, *Vulpes lagopus*.
Sources: [9,11,26,33,47–50].

33. Site: Koziarnia Cave (MIS 3).
Canid species: *Canis lupus spelaeus*, *Canis lupus lupus*, *Vulpes vulpes*, *Vulpes lagopus*.
Sources: [9,11,34–35,37,51].

34. Site: Stajnia Cave (MIS 6-1).
Canid species: *Canis lupus spelaeus*, *Canis lupus lupus*, *Cuon alpinus europaeus*, *Vulpes vulpes*, *Vulpes lagopus*.
Sources: [23,52].

35. Site: Raj Cave (MIS 5-2).
Canid species: *Canis lupus lupus*, *Vulpes vulpes*, *Vulpes lagopus*.
Sources: [9,53].

36. Site: Łokietka Cave (MIS 5-2).
Canid species: *Canis lupus lupus*, *Vulpes vulpes*, *Vulpes lagopus*.
Sources: [26].

37. Site: Towarna Cave (MIS 5-1).
Canid species: *Canis lupus lupus*, *Vulpes vulpes*, *Vulpes lagopus*.
Sources: [54].

38. Site: Ciemna Cave (MIS 5-1).
Canid species: *Canis lupus lupus*, *Vulpes vulpes*, *Vulpes lagopus*.
Sources: [26,34,55–56].

23. Site: Biśnik Cave, layers 12-1 (MIS 5-1).
Canid species: *Canis lupus spelaeus*, *Canis lupus lupus*, *Cuon alpinus europaeus*, *Vulpes vulpes*, *Vulpes lagopus*, *Vulpes corsac*.
Sources: [23].

26. Site: Naciekowa Cave (MIS 5-3).
Canid species: *Canis lupus spelaeus*, *Vulpes vulpes*, *Vulpes lagopus*.
Sources: [23,44–45].

39. Site: Obok Wschodniej Cave (MIS 4-3).
Canid species: *Canis lupus spelaeus*, *Vulpes vulpes*, *Vulpes lagopus*, *Vulpes corsac*.
Sources: [44–45].

40. Site: Północna Duża Cave (MIS 4-2).
Canid species: *Canis lupus spelaeus*, *Vulpes lagopus*.
Sources: [44–45].

41. Site: Wschodnia Cave (MIS 4-2).
Canid species: *Canis lupus spelaeus*, *Vulpes vulpes*, *Vulpes lagopus*.
Sources: [23,44–45].

42. Site: Radochowska Cave (MIS 5-1).
Canid species: *Canis lupus spelaeus*, *Canis lupus lupus*, *Cuon alpinus europaeus*, *Vulpes vulpes*, *Vulpes lagopus*.
Sources: [20,44–45,58-62].

43. Site: Obłazowa 1 Cave (MIS 3-1).
Canid species: *Canis lupus lupus*, *Vulpes vulpes*, *Vulpes lagopus*.
Sources: [63–64].

44. Site: Skarszyn (MIS 3, 45-35 kyr)
Canid species: *Canis lupus spelaeus*.
Sources: [65].

45. Site: Wrocław-Hallera (MIS 3, 45-35 kyr).
Canid species: *Canis lupus spelaeus*.
Sources: [66–67].

46. Site: Kraków-Spadzista (MIS 3, 33-29 kyr).
Canid species: *Canis lupus lupus*, *Vulpes lagopus*.
Sources: [26,68–70].

47. Site: Malschwitz (MIS 3?).
Canid species: *Canis lupus spelaeus*.
Sources: [67,71].

48. Site: Cave no 4 on Mount Birów (MIS 3-1).
Canid species: *Canis lupus spelaeus*, *Vulpes lagopus*.
Sources: [72].

49. Site: Borsucza Cave (MIS 3-1).
Canid species: *Vulpes vulpes*, *Vulpes lagopus*.

Sources: this paper.

50. Site: Jaksice 2 (MIS 2, 29-25 kyr).
Canid species: *Vulpes vulpes*, *Vulpes lagopus*.
Sources: [73].

51. Site: Borsuka Cave (MIS 3-1, 30-6 kyr).
Canid species: *Canis lupus lupus*, *Vulpes vulpes*, *Vulpes lagopus*.
Sources: [74].

52. Site: Pietraszyn (MIS 3).
Canid species: *Canis lupus spelaeus*, *Vulpes lagopus*.
Sources: this paper.

53. Site: Pyskowice (MIS 3).
Canid species: *Canis lupus spelaeus*, *Vulpes lagopus*.
Sources: this paper.

54. Site: Obłazowa 2 (MIS 3, 35-30 kyr).
Canid species: *Vulpes vulpes*.
Sources: [75].

55. Site: Biały Kamień Cave (MIS 3-1).
Canid species: *Canis lupus lupus*.
Sources: [44–45].

56. Site: Niedźwiedzia Górna Cave (MIS 3-1).
Canid species: *Canis lupus lupus*, *Vulpes vulpes*, *Vulpes lagopus*.
Sources: this paper.

57. Site: Zegar Cave (MIS 3-1).
Canid species: *Canis lupus lupus*, *Vulpes vulpes*, *Vulpes lagopus*.
Sources: [76].

58. Site: Miniaturka Cave (MIS 3-1).
Canid species: *Vulpes vulpes*, *Vulpes lagopus*.
Sources: [44–45].

59. Site: Rogóżka Cave (MIS 3-1).
Canid species: *Canis lupus lupus*, *Cuon alpinus europaeus*, *Vulpes vulpes*, *Vulpes lagopus*, *Vulpes corsac*.
Sources: [23,44–45,77–78].

60. Site: Zbójecka Cave (MIS 3-1).
Canid species: *Canis lupus lupus*, *Vulpes vulpes*, *Vulpes lagopus*.
Sources: [2,34–35].

61. Site: Solna Jama Cave (MIS 3-1).
Canid species: *Canis lupus lupus*, *Vulpes vulpes*, *Vulpes lagopus*.
Sources: [23,44–45,79].

62. Site: Magurska Cave (MIS 3-1).
Canid species: *Canis lupus lupus*.
Sources: [32–33,80–81].

63. Site: Gorenicka Cave (MIS 3-1).
Canid species: *Canis lupus lupus*, *Vulpes vulpes*.

Sources: [35].

64. Site: Deszczowa Cave, fauna 2 (MIS 3-1).
Canid species: *Canis lupus lupus*, *Vulpes vulpes*, *Vulpes lagopus*.
Sources: [25–27].
65. Site: Wilczyce (MIS 2, 16.1-15.5 kyr).
Canid species: *Vulpes vulpes*, *Vulpes lagopus*.
Sources: [82–86].

66. Site: Żytnia Skała Shelter (MIS 2-1).
Canid species: *Canis lupus lupus*, *Vulpes vulpes*, *Vulpes lagopus*.
Sources: [9,87].

67. Site: Murek Cave (MIS 2-1).
Canid species: *Vulpes lagopus*.
Sources: this paper.

68. Site: Nad Potoczkiem Cave (MIS 2-1).
Canid species: *Vulpes lagopus*.
Sources: this paper.

69. Site: Jasna Strzegowska Cave (MIS 2-1).
Canid species: *Vulpes lagopus*.
Sources: this paper.

70. Site: Zygmuntówka Quarry (MIS 2).
Canid species: *Canis lupus, Vulpes lagopus*.
Sources: this paper.
71. Site: Ruska Skała Shelter (MIS 2-1).
Canid species: *Vulpes lagopus*.
Sources: [88].

72. Site: Cisowe 1 Shelter (MIS 2-1).
Canid species: *Canis lupus lupus*, *Vulpes vulpes*, *Vulpes lagopus*.
Sources: [44–45].

73. Site: Na Ścianie Cave (MIS 2-1).
Canid species: *Canis lupus lupus*, *Vulpes vulpes*.
Sources: [44-45].

74. Site: Aven w Połomie Cave (MIS 2-1).
Canid species: *Canis lupus lupus*, *Vulpes vulpes*.
Sources: [44–45].

75. Site: Podlesice Shelter (MIS 2-1, 16.5-10.5 kyr).
Canid species: *Vulpes lagopus*.
Sources: [11,88–89].

76. Site: Okiennik Cave (MIS 2-1).
Canid species: *Canis lupus lupus*, *Vulpes vulpes*, *Vulpes lagopus*.
Sources: [2,34,55,90].

77. Site: na Miłaszówce Cave (MIS 2-1).
Canid species: *Canis lupus lupus*.
Sources: [31,91].

78. Site: na Gołąbcu Cave (MIS 2-1).
Canid species: *Canis lupus lupus*, *Vulpes lagopus*.
Sources: [92–93].

79. Site: Krucza Skała Shelter (MIS 2-1, 16.5-13 kyr).
Canid species: *Vulpes* sp., *Vulpes lagopus*.
Sources: [94].

80. Site: Maszycka Cave (MIS 2-1).
Canid species: *Vulpes vulpes*.
Sources: [34,95].

81. Site: Sąspowska Zachodnia Cave (MIS 1).
Canid species: *Canis lupus lupus*, *Vulpes vulpes*.
Sources: [96].

82. Site: in Sobczański Wąwóz Cave (MIS 1).
Canid species: *Vulpes vulpes*.
Sources: [97].

83. Site: Puchacza Skała Shelter (MIS 1).
Canid species: *Canis lupus lupus*, *Vulpes vulpes*, *Vulpes lagopus*.
Sources: [9,98].

84. Site: Mroczna w Pośrednicy Cave (MIS 1).
Canid species: *Canis lupus lupus*, *Vulpes vulpes*.
Sources: [99].

85. Site: Shelter in Pośrednica 2 (MIS 1).
Canid species: *Vulpes vulpes*.
Sources: [100].

86. Site: Bramka Shelter (MIS 1).
Canid species: *Canis lupus lupus*, *Vulpes vulpes*, *Vulpes lagopus*.
Sources: [101–102].

87. Site: nad Galoską Cave (MIS 1).
Canid species: *Canis lupus lupus*, *Vulpes vulpes*.
Sources: [34,103].

88. Site: na Gaiku 2 Shelter (MIS 1).
Canid species: *Vulpes vulpes*.
Sources: [34,104].

89. Site: Przegińska Cave (MIS 1).
Canid species: *Vulpes vulpes*.
Sources: [34,103–104].

90. Site: Poszukiwaczy Skarbów Cave (MIS 1).
Canid species: *Canis lupus lupus*.
Sources: [93,105].

91. Site: Duża Cave at Mączna Skała (MIS 1).
Canid species: *Vulpes vulpes*.
Sources: this paper.

92. Site: Zalas Shelter (MIS 1).
Canid species: *Canis lupus lupus*, *Vulpes vulpes*.
Sources: [106].

93. Site: Cisowe 2 Shelter (MIS 1).
Canid species: *Vulpes vulpes*, *Vulpes lagopus*.
Sources: [44–45].

94. Site: Małgorzaty Shelter (MIS 1).
Canid species: *Canis lupus lupus*, *Vulpes vulpes*.
Sources: [44–45].

95. Site: Kuny Shelter (MIS 1).
Canid species: *Canis lupus lupus*, *Vulpes vulpes*.
Sources: [45].

96. Site: Wilcze Shelter (MIS 1).
Canid species: *Canis lupus lupus*, *Vulpes vulpes*.
Sources: this paper.

97. Site: Górne Shelter above Stajnia Cave (MIS 1).
Canid species: *Canis lupus lupus*, *Vulpes vulpes*.
Sources: this paper.

98. Site: Babie Nogi Shelter (MIS 1).
Canid species: *Canis lupus lupus*, *Vulpes vulpes*.
Sources: this paper.

99. Site: Panna Shelter (MIS 1).
Canid species: *Vulpes vulpes*.
Sources: [44–45].

100. Site: Trwoga Paleontologa Shelter (MIS 1).
Canid species: *Canis lupus lupus*, *Vulpes vulpes*.
Sources: [44–45].

101. Site: Pośrednie Shelter (MIS 1).
Canid species: *Vulpes vulpes*.
Sources: [100].

102. Site: Wilcze 1 Shelter (MIS 1).
Canid species: *Vulpes vulpes*.
Sources: [99].

103. Site: Shelter above Niedostępna Cave (MIS 1).
Canid species: *Vulpes vulpes*.
Sources: [96].

104. Site: Ciasna Cave (MIS 1).
Canid species: *Vulpes vulpes*.
Sources: this paper.

105. Site: nad Mosurem Starym Duża Cave (MIS 1, 3.5-1.5 kyr).
Canid species: *Vulpes vulpes*.
Sources: [11].

106. Site: Kontaktowa Cave (MIS 1).
Canid species: *Vulpes vulpes*.
Sources: [44–45].

107. Site: Jasna Smoleńska Cave (MIS 1).
Canid species: *Vulpes vulpes*.
Sources: [96].

108. Site: Olsztyńska i Wszystkich Świętych Cave (MIS 1).
Canid species: *Vulpes vulpes*.
Sources: [97].

109. Site: Ostrężnicka Cave (MIS 1).
Canid species: *Vulpes vulpes*.
Sources: [96].

110. Site: Kryształowa Cave (MIS 1).
Canid species: *Vulpes vulpes*.
Sources: this paper.

111. Site: Small Przy Torach Cave (MIS 1).
Canid species: *Vulpes vulpes*.
Sources: [44–45].

112. Site: Tczew (?).
Canid species: *Canis lupus* ssp.
Sources: [2,107].

113. Site: Elbląg (?).
Canid species: *Canis lupus* ssp.
Sources: [2,108–111].

114. Site: Trzebowisko (?).
Canid species: *Vulpes vulpes*.
Sources: [112].

115. Site: Rzeszów, Wisłoka river (?).
Canid species: *Canis lupus lupus*.
Sources: [33,112].

116. Site: Wielopolka (?).
Canid species: *Canis lupus* ssp.
Sources: [112].

References

1. Stach, J. *Nyctereutes* (Canidae) w pliocenie Polski. *Acta Geol. Pol.* **1954**, *4(2)*, 191–206.

2. Kowalski, K. *Katalog ssaków plejstocenu Polski*. PWN, Warszawa, 1959; pp 227.

3. Kowalski, K. Les mammiferes fossiles des remplissages karstiques de Pologne - prohlemes paleobiologiques et stratigraphiques. In *Livre du centenaire Emile G. Racovitza, 1868-1968*; Orghidan, T.; Racovitza, G., Ed.; Académie de la république socialiste de Roumanie, Bucarest, **1970**, pp 491–498.

4. Kowalski, K. Historia i ewolucja lądowej fauny Polski (red.). *Fol. Quat.* **1989a**, *59-60*, 1–276.

5. Kowalski, K. Stratigraphy of Neogene mammals of Poland. In *European Neogene mammal chronology.* Lindsay, E.H.; Fahlbusch, V.; Mein P., Eds.; Plenum Press, New York, **1990**, pp 193–209.

6. Czyżewska, T. *Nyctereutes sinensis* Schlosser (Canidae, Mammalia) from the Pliocene Breccia in Węże (Poland). *Acta Zool. Crac.* **1969**, *14(17)*, 441–450.

7. Czyżewska, T. Natural endocranial casts of the Canidae from Węże I near Działoszyn (Poland). *Acta Zool. Crac.* **1981**, *25(9)*, 251–260.

8. Ivanoff, D.V.; Wolsan, M.; Marciszak, A. 2014. Brainy stuff of long-gone dogs: a reappraisal of the supposed *Canis* endocranial cast from the Pliocene of Poland. Sci. Nat. **2014**, *101(8)*, 645-651. [https://doi.org/10.1007/s00114-014-1200-4]

9. Wolsan, M. Drapieżne - Carnivora. *Fol. Quat.* **1989**, *59-60*, 177–197.

10. Wolsan, M. Lower Pleistocene carnivores of Poland. *Quarterpäläntologie* **1990**, *8*, 277–280.

11. Wolsan, M. Evolution des carnivores quaternaires en Europe centrale dans leur contexte stratigraphique et paleoclimatique. *L'Anthropologie* **1993**, *97*, 203–222.

12. Bosák, P.; Głazek, J.; Horácek, I.; Szynkiewicz, A. New locality of Early Pleistocene vertebrates - Żabia Cave at Podlesice, Central Poland. *Acta Geol. Pol.* **1982**, *32(3-4)*, 217–226.

13. Nadachowski, A.; Stefaniak, K.; Szynkiewicz, A.; Marciszak, A.; Socha, P.; Schick, P.; August, C. Biostratigraphic importance of the Early Pleistocene fauna from Żabia Cave (Poland) in Central Europe. *Quat. Int.* **2011**, *243(1)*, 204–218. [https://doi.org/10.1016/j.quaint.2011.04.037]

14. Wiszniowska, T. Middle Pleistocene Carnivora (Mammalia) from Kozi Grzbiet in the Świętokrzyskie Mts., Poland. *Acta Zool. Crac.* **1989a**, *32*, 589–630.

15. Langenhan, A. Ueber fossile Funde am Kitzelberg. *Z. Dtsch. Geol. Ges.* **1904a**, *56*, 5–7.

16. Langenhan, A. Schädel eines Höhlenwolfs im Kitzelberg bei Kauffung im Katzbachtal. *Monatssch. Min., Gest.- und Petrefakt* **1904b**, *2*, 24–25.

17. Langenhan, A. Funde fossiler Tierreste am Kitzelberg bei Kauffung im Katzbachtal. *Monatssch. Min., Gest.- und Petrefakt* **1904c**, *2*, 26–28.

18. Langenhan, A. Über neue Funde fossiler Tierarten am Kitzelberg bei Kauffung. *Wanderer im Riesengebirge* **1904d**, *24(4/258)*, 56–58.

19. Wenke, R. Die diluvialen Säugetierreste vom Kitzelberge bei Kauffung. Wanderer in Riesengebirge, **1933**, *53 (9)*, 146–147.

20. Zotz, L.F. Die Altsteinzeit in Niederschlesien. Kabitsch Verlag, Leipzig, **1939a**, pp 144.

21. Zotz, L.F. Zur Altsteinzeit in Niederschlesien. *Altsch. Bl.* **1939b**, *14 (3)*, 94–98.

22. Kot, M.; Berto, C.; Krajcarz, M.T.; Moskal-del Hoyo, M.; Gryczewska, N.; Szymanek, M.; Marciszak, A.; Stefaniak, K.; Zarzecka-Szubińska, K.; Lipecki, G.; Wertz, K.; Madeyska, T. Frontiers of the Lower Palaeolithic expansion in Europe: Tunel Wielki Cave (Poland). *Sci. Rep.* **2022**, *12*, 16355. [https://doi.org/10.1038/s41598-022-20582-0]
23. Marciszak, A.; Kropczyk, A.; Lipecki, G. The first record of *Cuon alpinus* (Pallas, 1811) from Poland and the possible impact of other large canids on the evolution of the species. *J. Quat. Sci.* **2021**, *36(6)*, 1101–1121. [https://doi.org/10.1002/jqs.3340]
24. Marciszak, A.; Socha, P.; Nadachowski, A.; Stefaniak, K. Carnivores from Biśnik Cave. *Quat. Hors-serie* **2011**, *4*, 101–106.
25. Cyrek, K.; Nadachowski, A.; Madeyska, T.; Bocheński, Z.; Tomek, T.; Wojtal, P.; Miękinia, B.; Lipecki, G.; Garapich, A.; Rzebik-Kowalska, B.; Stworzewicz, E.; Wolsan, M.; Godawa, J.; Kościów, R.; Fostowicz-Frelik, Ł.; Szyndlar, Z. Excavation in the Deszczowa Cave (Kroczyckie Rocks, Czestochowa Upland, Central Poland). *Folia Quat*. **2000**, *71*, 5–84.
26. Wojtal, P. Zooarchaeological studies of the Late Pleistocene sites in Poland. ISEA PAS, Cracow, 2007, pp 189.
27. Nadachowski, A.; Żarski, M.; Urbanowski, M.; Wojtal, P.; Miękina, B.; Lipecki, G.; Ochman, K.; Krawczyk, M.; Jakubowski, G.; Tomek, T. Late Pleistocene environment of the Częstochowa Upland (Poland) reconstructed on the basis of faunistic evidence from archaeological cave sites. *ISEA PAS,* Kraków, 2009, pp 112.
28. Made v. d. J.; Stefaniak, K.; Marciszak, A. The Polish fossil record of the wolf *Canis* and the deer *Alces*, *Capreolus*, *Megaloceros*, *Dama* and *Cervus* in an evolutionary perspective. *Quat. Int.* **2014**, *326-327*, 406–430. [https://doi.org/10.1016/j.quaint.2013.11.015]
29. Ossowski, G. Sprawozdanie z badań paleo-etnologicznych w jaskiniach okolic Ojcowa w r. 1886. *Zbiór Wiad. Antrop. Kraj.***1887a**, *11*, 12–32.
30. Ossowski, G. Fouillos de la Caverne de Wierzchowska-Gorna en Pologne. *Antiqua* **1887b,** *2(2-5)*, 37–47.
31. Woldřich, J. N. Diluviale europäisch-nordasiatische Säugethierfauna und ihre Beziehungen zum Menschen. *Mém. Acad. imp. sci. St.-Pétersbg. hist. Acad*. **1887**, *35(10)*, 1–162
32. Kiernik, E. Materiały do paleozoologii dyluwialnych ssaków ziem polskich. Cz. 5. Szczątki wilka z dyluwium warstw ziem polskich. *Roz. Wydz. Mat.-Przyrod. PAU III* **1913a**, *13*, 491–541.
33. Kiernik, E. Materiały do paleozoologii dyluwialnych ssaków ziem polskich. Cz. 5. Familia: Canidae; Sectio: Lupinae. *Spraw. czynn. pos. PAU Kraków* **1913b**, *18(7)*, 15.
34. Kowalski, K. *Jaskinie Polski, tom 1.* Państwowe Muzeum Archeologiczne, Warszawa, **1951**; p 466.
35. Römer, F. Die Knochenhöhlen von Ojców in Polen. *Palaeontographica* **1883**, *29*, 1–41.
36. Kowalski, K. *Jaskinie Polski.* Wiedza Powszechna, seria: Przyroda Polska. Warszawa, **1965**; p 144.
37. Bigaj, J. Szczątki Canidae z plejstocenu Polski. *Folia Quat.* **1963**, *13*, 1–18.
38. Wiszniowska, T. Nowe znalezisko paleontologiczne w Sudetach. *Przeg. Zool.* **1967**, *11*, 430–433.
39. Wiszniowska, T. Wstępne wyniki badań fauny kopalnej w Jaskini Niedźwiedziej. *Acta Univ. Wratislav.* **1970**, *127*, 45–70.
40. Wiszniowska, T. Szczątki fauny w namuliskach jaskiń na Śląsku. *Prace Kom. Arch. PAN* **1986**, *3*, 9–19.

41. Wiszniowska, T. Kopalne szczątki zwierzęce. In *Jaskinia Niedźwiedzia w Kletnie. Badania i udostępniani;* Jahn, A.; Kozłowski, S.; Wiszniowska, T., Eds.; Zakład Narodowy imienia Ossolińskich, Wrocław-Warszawa, 1989b, pp 255–279.
42. Stefaniak, K.; Bieroński, J. Caves and rock shelters of the Eastern Sudetes - catalogue of important speleological features. *Stud. Fac. Earth Scien. Univ. Silesia* **2009**, *56*, 515–534.
43. Bieroński, J.; Stefaniak, K.; Hercman, H.; Socha, P.; Nadachowski, A. Palaeogeographical and palaeoecological studies of sediments of the Niedźwiedzia (Bear) Cave in Kletno. *Stud. Fac. Earth Scien. Univ. Silesia* **2009c**, *56*, 401–422.
44. Marciszak, A.; Stefaniak, K.; Gornig, W. Fossil theriofauna from the Sudety Mts (SW Poland). *The state of research. Cranium* **2016**, *33(1)*, 31–41.
45. Marciszak, A.; Sobczyk, A.; Kasprzak, M.; Gornig, W.; Ratajczak, U.; Wiśniewski, A.; Stefaniak K. Taphonomic and paleoecological aspects of large mammals from Sudety Mts (Silesia, SW Poland), with particular interest to the carnivores. *Quat. Int.* **2020**, *546*, 42–63. [https://doi.org/10.1016/j.quaint.2019.11.009]
46. Chmielewski, W. Stanowisko paleolityczne w Dziadowej Skale koło Skarżyc w pow. zawierciańskim. *Prace Mat. Muzeum Archeol. Etnogr. Łódź Ser. Archeologiczna* **1958**, *3*, 5–48.
47. Zawisza, J. Jaskinia Mamuta w Dolinie Wierzchowskiej. *Wiad. Archeol.* **1874**, *2*, 5–23.
48. Zawisza J. Dalsze poszukiwania w Jaskini Mamuta w czerwcu 1874 oraz Jaskinia Okopy odkryta w 1874. *Wiad. Archeol.* **1876**, *3*, 125–139.
49. Zawisza J. La caverne de Mammouth en Pologne. *Mém. Soc. géol. Fr.* **1878**, *1(2)*, 439–447.
50. Lubicz-Niezabitowski, E. Pardwy *Lagopus lagopus* L. i *Lagopus mutus* Montin oraz towarzysząca im fauna w czwartorzędzie Polski. *Rocz. Pol. Tow. Geol.* **1932**, *7(2)*, 179–192.
51. Chmielewski, W.; Kowalski, K.; Madeyska-Niklewska, T.; Sych, L. Wyniki badań osadów jaskini Koziarni w Sąspowie, pow. Olkusz. *Folia Quat*. **1967**, *26*, 1–69
52. Żarski, M.; Winter, H.; Nadachowski, A.; Urbanowski, M.; Socha, P.; Kenig, K.; Marcinkowski, B.; Krzemińska, E.; Stefaniak, K.; Nowaczewska, W.; Marciszak, A. Stratigraphy and palaeoenvironment of Stajnia Cave (southern Poland) with regard to habitation of the site by Neanderthals. *Geological Quarterly* **2017**, *61(2)*, 350–369. [https://doi.org/10.7306/gq.1355]
53. Kowalski, K. Fossil fauna. Studies on Raj Cave near Kielce (Poland) and its deposits. *Fol. Quat.* **1972**, 41, 45–60.
54. Kopacz, J.; Skalski, A.W. Excavations of the cave system in the Towarne Mountains near Częstochowa. *Arch. Pol.* **1976**, *17*, 163–175.
55. Krukowski, S. Paleolit. Prehistoria Ziem Polskich, Kraków, **1939**, 4, cz. 1.
56. Kowalski, K. *Jaskinie Polski, tom III*. Państwowe Wydawnictwo Naukowe, Warszawa, **1954**; p 191.
57. Marciszak, A. Carnivores from Naciekowa Cave (Połom Hill, Kaczawskie Mts., south-west Poland). In *Guidebook of the 17th International Cave Bear Conference, September 15th-18th 2011*; Döppes, D.; Jogerm, U.; Nielbock, R.; Rosendahl W., Eds.; Einhornhöhle, Harz, Germany, 2011, p 20.
58. Frenzel, J. Knochenfunde in der Reyersdorfer Tropsteinhöhle. *Beit. Biol. Glatzer Schneeberges* **1936**, *2*, 121–134.

59. Frenzel, J. Die Reyersdorfer Tropsteinhöhle, ein schlesisches Naturdenkmal. *Schles. Heimat* **1937**, *1*, 6–11.
60. Bieroński, J.; Burdukiewicz, J.M.; Wiszniowska, T. Wyniki nowych badań Jaskini Radochowskiej. *Śląskie Spraw. Archeol.* **1985**, *26*, 5–18.
61. Bieroński, J.; Socha, P.; Stefaniak, K. Deposits and fauna of the Sudeten caves - the state of research. *Stud. Fac. Earth Scien. Univ. Silesia* **2007**, *45*, 183–201.
62. Bieroński, J.; Burdukiewicz, J.M.; Socha, P.; Stefaniak, K.; Hercman, H.; Nadachowski, A. Palaeogeographical, archeological and palaeozoological studies in the Radochowska Cave. *Stud. Fac. Earth Scien. Univ. Silesia* **2009a**, *56*, 455–475.
63. Lipecki, G.; Wolsan M. Carnivorans (Carnivora). In *Obłazowa Cave: human activity, stratigraphy and palaeoenvironment*; Valde-Nowak, P.; Nadachowski, A.; Madeyska, T., Eds.; IAE PAS, Kraków, **2003**, pp 125–129.
64. Valde-Nowak, P.; Nadachowski, A. Micoquian assemblage and environmental conditions for the Neanderthals in Obłazowa Cave, Western Carpathians, Poland. *Quat. Int.* **2014**, *326-327*, 146–156. [https://doi.org/10.1016/j.quaint.2013.08.057]
65. Kropczyk, A.; Marciszak, A. Ssaki stepu mamuciego ze śląskiego stanowiska Skarszyn koło Wrocławia. Kopernikańskie Seminarium Doktoranckie, Uniwersytet Mikołaja Kopernika w Toruniu, Toruń. Monografia pokonferencyjna XV, **2022** (in press).
66. Wiśniewski, A.; Stefaniak, K.; Wojtal, P.; Zych, J.; Nadachowski, A.; Musil, R.; Badura, J.; Przybylski, B. Archaeofauna or palaeontological record? Remarks on Pleistocene fauna from Silesia. *Spraw. Archeol.* **2009**, *61*, 1–62.
67. Marciszak, A.; Kotowski, A.; Przybylski, B.; Badura, J.; Wiśniewski, A.; Stefaniak, K. Large mammals from historical collections of open-air sites of Silesia (southern Poland) with special reference to carnivores and rhinoceros. *Hist. Biol.* **2019a**, *31(6)*, 696–730. [https://doi.org/10.1080/08912963.2017.1388377]
68. Lipecki, G.; Wojtal, P. Carnivores from the open-air Gravettian site Kraków Spadzista. In *A Gravettian site in Southern Poland: Kraków Spadzista;* Wilczyński, J. Wojtal, P. Haynes G., Eds.; ISEA PAS, Kraków, **2015**, pp 117–157.
69. Wilczyński, J.; Wojtal, P.; Sobczyk, K. Spatial organization of the Gravettian mammoth hunters site – Kraków Spadzista (southern Poland). *J. Archaeol. Sci.* **2012**, *39*, 3627–3642. [https://doi.org/10.1016/j.jas.2012.05.012]
70. Wojtal, P.; Wilczyński, J. Zooarchaeological studies of large mammal remains from Kraków Spadzista site - trench C2 and trench E1 (2011-2012 excavations). In *A Gravettian site in southern Poland: Kraków Spadzista*; Wilczyński, J.; Wojtal, P.; Haynes, G., Eds.; ISEA PAS, Kraków, 2015, pp 93–111.
71. Herr, O. Diluviale und altalluviale Säugetierreste aus der Oberlausitz. *Abh. Naturforsch. Ges. Görlitz* **1924**, *29*, 92–101.
72. Muzolf, B.; Stefaniak, K.; Tomek, T.; Wertz, K.; Socha, P.; Cyrek, K.; Mirosław-Grabowska, J.; Madeyska, T.; Nadachowski, A. Multiculturate sites on Mt. Birów in Podzamcze. *Stud. Fac. Earth Scien. Univ. Silesia* **2009**, *56*, 283–294.

73. Wilczyński, J.; Wojtal, P.; Łanczont, M.; Mroczek, P.; Sobieraj, D.; Fedorowicz, S. Loess, flints and bones: Multidisciplinary research at Jaksice II Gravettian site (southern Poland). *Quat. Int.* **2015**, *359-360*, 114–130. [https://doi.org/10.1016/j.quaint.2014.04.002]
74. Marciszak, A.; Lipecki, G.; Gornig, W.; Wilczyński, J. Carnivores from the Borsuka Cave (southern Poland) as an example of changes in carnivore assemblages during MIS 2 and MIS 1. *Acta Zool. Crac.* **2017**, *A60(2)*, 105–146. [DOI: 10.3409/azc.60_2.105.]
75. Nadachowski, A.; Harrison, D.L.; Szyndlar, Z.; Tomek, T.; Wolsan, M. Late Pleistocene vertebrate fauna from Obłazowa 2 (Carpathians, Poland): palaeological reconstruction. *Acta Zool. Crac.* **1993**, *36(2)*, 281–290.
76. Stefaniak, K.; Muzolf, B.; Mirosław-Grabowska, J.; Socha, P. Studies in the caves of the Zegarowe Rocks. In *Karst of the Częstochowa Upland and the Eastern Sudetes–palaeoenvironments and protection. Stud. Fac. Earth Scien. Univ. Silesia, 56*; Stefaniak, K.; Socha, P.; Tyc, A., Eds.; 2009a, pp 255–271.
77. Pax, F.; Maschke, K. Höhlenfauna des Glatzer Schneeberges. Die rezente Metazoenfauna. *Beit. Biol. Glatzer Schneeberges* **1935**, *1*, 4–72.
78. Bieroński, J.; Socha, P.; Stefaniak, K.; Hercman, H.; Gąsiorowski, M. Caves in Rogóżka-origin, sediments and fauna. *Stud. Fac. Earth Scien. Univ. Silesia* **2009b**, *56*, 477–489.
79. Marciszak, A.; Gornig, W.; Stefaniak, K. Large mammals (carnivores, artiodactyls) from Solna Jama Cave (Bystrzyckie Mts, Southwestern Poland) in the context of faunal changes in the postglacial period of Central Europe. *Palaeontol. Electron.* **2017**, *20(3A)*, 1–37. [https://doi.org/10.26879/581]
80. Lubicz-Niezabitowski, E. O kilku ciekawszych szczątkach kopalnych zwierząt ssących Polski. *Kosmos A* **1938a**, *62*, 431–438.
81. Lubicz-Niezabitowski, E. O kilku ciekawszych szczątkach kopalnych zwierząt ssących Polski. *Spraw. Pozn. TPN. Wydz. Mat.-Przyr.* **1938b**, *11*, 77–78.
82. Bratlund, B. 2002. The faunal remains from Wilczyce. Recent studies on the final Palaeolithic of the European plain. In *Proceedings of a U.I.S.P.P. Symposium, Stockholm. Jutland Archaeological Society Publications 39*; Eriksen, B.V.; Bratlund B., Ed.; Narayana Press: Højbjerg. **1999**, 101–107
83. Irish, J.D.; Bratlund, B.; Schild, R.; Kolstrup, E.; Królik, H.; Mańka, D.; Boroń, T. A late Magdalenian perinatal human skeleton from Wilczyce, Poland. *J. Hum. Evol.* **2008**, *55*, 736–740. [https://doi.org/10.1016/j.jhevol.2008.03.007]
84 Lasota-Moskalewska, A. Wilczyce. A Late Magdalenian winter hunting camp in southern Poland. In *Faunal remains;* Schild, R., Ed.; IAE PAS, Warszawa, **2014**, pp 105–118.
85. Nadachowski, A.; Bratlund, B.; Tomek, T.; Miękinia, B.; Stworzewicz, E.; Szyndlar, Z. Faunal remains from Wilczyce and the paleoecological reconstruction of the area at the end of the Pleniglacial. In *Wilczyce. A Late Magdalenian winter hunting camp in southern Poland*; Schild, R., Ed.; IAE PAS, Warszawa, **2014**, pp 119–134.
86. Schild, R. Taphonomy and chronology of the settlement. In *Wilczyce. A Late Magdalenian winter hunting camp in southern Poland*; Schild R., Ed.; IAE PAS, Warszawa, 2014, pp 87–104.
87. Kowalski, K.; Kozłowski, J. K.; Krysowska-Iwaszkiewicz, M.; Pawlikowa, B.; Wiktor, A. Badania osadów schronisk podskalnych w Żytniej Skale (Bębło, pow. Kraków). *Fol. Quat.* **1967**, *25*, 1–48.

88. Krysiak, K. Szczątki zwierzęce ze schroniska skalnego pod Podlesicami w pow. zawierciańskim. *Prace Mat. Muzeum Archeol. Etnogr. Łódź Ser. Archeologiczna*, **1956**, 41–47.
89. Chmielewska, M.; Pierzchałko L. Stanowisko wczesnomezolityczne w schronisku skalnym koło Podlesic w pow. zawierciańskim. *Prace Mat. Muzeum Archeol. Etnogr. Łódź Ser. Archeologiczna* **1956**, *1*, 29–40.
90. Demetrykiewicz, Z.; Kuźniar, W. Najstarszy paleolit na ziemiach polskich oraz inne wykopaliska odkryte w jaskini "Okiennik". *Prace Mat. Muzeum Archeol. Etnogr. Łódź Ser. Archeologiczna* **1914**, *13*, 10–43.
91. Ossowski, G. Czwarte sprawozdanie z badań antropologiczno-archeologicznych w jaskiniach okolic Krakowa w 1882. *Zbiór Wiad. Antrop. Kraj.* **1883b**, *7*, 66–88.
92. Ossowski, G. Sprawozdanie z badań geologiczno–antropologicznych dokonanych w 1879 w jaskiniach okolic Krakowa. *Zbiór Wiad. Antrop. Kraj.* **1880**, *4*, 35–57.
93. Kowalski, K. *Jaskinie Polski, tom II* . Państwowe Muzeum Archeologiczne, Warszawa, **1953**; p 186.
94. Nadachowski, A.; Krajcarz, M.; Lemanik, A.; Lipecki, G.; Marciszak, A.; Miękina, B.; Stefaniak, K.; Tomek, T. Vertebrate remains from Krucza Skała Rockshelter. In *Late Magdalenian Campsite at Krucza Skała Rockshelter*; Cyrek, K.; Sudoł-Procyk, M.; Czyżewski, Ł., Eds.; Wyd. Nauk. UMK, Toruń, **2022**, pp 138–160.
95. Ossowski, G. Jaskinie okolic Ojcowa pod względem paleo-etnologicznym. *Pam. PAU Wyd. Mat.-Przyr.* **1885**, *11*, 1–60.
96. Madeyska, T. Środowisko człowieka w środkowym i górnym paleolicie na ziemiach Polskich w świetle badań geologicznych. *Studia Geol. Pol.* **1981**, *69*, 7–125.
97. Aleksandrowicz, S.W.; Nadachowski, A.; Rydlewski, J.; Valde-Nowak, P.; Wołoszyn, B.W. Subfossil fauna from a cave in the Sobczański Gully (Pieniny Mts., Poland). *Folia Quat.* **1985**, *56*, 57–78.
98. Kowalski, K.; Kozłowski, J. K.; Krysowska, M.; Wiktor, A. Badania osadów schroniska w Puchaczej Skale w Prądniku Czerwonym, powiat Olkusz. *Fol. Quat.* **1965**, *20*, 1–44.
99. Stefaniak, K.; Nadachowski, A.; Tomek, T.; Socha, P. Palaeontological studies in the Częstochowa Upland. In *Karst of the Częstochowa Upland and the Eastern Sudetes–palaeoenvironments and protection. Stud. Fac. Earth Scien. Univ. Silesia 56*; Stefaniak K., Socha P., Tyc A. Eds.; 2009b, pp 85–144.
100. Stefaniak, K.; Socha, P.; Tyc, A.; Cyrek, K.; Nadachowski, A. Caves, rock shelters and palaeontological sites in quarries of the Częstochowa Upland - cataloque of important speleological features. In *Karst of the Częstochowa Upland and the Eastern Sudetes - palaeoenvironments and protection. Stud. Fac. Earth Scien. Univ. Silesia 56;* Stefaniak, K.; Socha, P.; Tyc, A., Eds.; 2009c, pp 307–354.
101. Nadachowski, A. Fauna kopalna płazów, gadów i ssaków w osadach jaskiń i schronisk Doliny Sąspowskiej. In *Jaskinie Doliny Sąspowskiej. Tło przyrodnicze osadnictwa pradziejowego*. Prace IA UW, WUW, Warszawa, Chmielewski, W. Ed.; 1988a, pp 19-39.
102. Nadachowski, A. Osady jaskiń i schronisk Doliny Saspowskiej. In *Jaskinie Doliny Sąspowskiej. Tło przyrodnicze osadnictwa pradziejowego*. Prace IA UW, WUW, Warszawa, Chmielewski, W. Ed.; 1988b, pp 77-173.
103. Ossowski, G. Drugie sprawozdanie z badań geologiczno-antropologicznych w jaskiniach okolic Krakowa w r. 1880. *Zbiór Wiad. Antrop. Kraj.* **1881**, *5*, 18–46.

104. Ossowski, G. O szczątkach fauny dyluwialnej znalezionych w namule jaskiń wąwozu mnikowskiego. *Sprawozdanie Komisji Fizjograficznej* **1883a**, *17*, 91–103.
105. Hoyer, H.F. Wyniki badan kości zebranych w 1922 w grotach dol. Kościeliskiej. *Spraw. Kom. Fizjog.* **1928**, *62*, 27–30.
106. Bocheński, Z.; Ginter, B.; Kozłowski, J. K.; Mook W. G.; Muszyński, M.; Nadachowski, A.; Stworzewicz, E.; Szyndlar, Z. Badania osadów jaskiniowych schronisk w Zalasie koło Krakowa. *Folia Quat*. **1985**, *56*, 15–37.
107. Conwentz, H. *Amtlicher Bericht über die Verwaltung der naturhistorischen, archaeologischen und ethnologischen Sammlungen des Westpreussischen Provinzial Museums für das Jahr 1894*, Danzig, **1899**, p 35,
108. Schirmacher, E. Die diluvialen Wirbelthierreste der Provinz Ost- und Westpreussen. Inaugural-Dissertation, Konigsberg, 1882, pp 135.
109. Jentzsch, A. Über den Seehund des Elbinger Yoldia–Thones. *Z. Dtsch. Geol. Ges.* **1928**, *39*, 496–498.
110. Hermann, R. Die Rhinocerosarten des westpreussischen Diluviums. *Sch. Naturf. Gesellsch. Danzig, NF.* **1913**, *13*, 110–173.
111. Sonntag, P. Geologie von Westpreussen. Berlin, Borntraeger, 1919, 240 p.
112. Friedberg, W. Atlas geologiczny Galicji. *Kom. Fizjog. Akademii Umiej*. **1903**, *1(16)*, 1–147.
